# Efficacy and Safety of Intranasal Esketamine in Patients With Treatment-Resistant Depression and Comorbid Chronic Post-traumatic Stress Disorder: Open-Label Single-Arm Pilot Study

**DOI:** 10.3389/fpsyt.2022.865466

**Published:** 2022-07-08

**Authors:** Maud Rothärmel, Cherifa Benosman, Wissam El-Hage, Caroline Berjamin, Diane Ribayrol, Olivier Guillin, Raphaël Gaillard, Lucie Berkovitch, Virginie Moulier

**Affiliations:** ^1^Service Hospitalo-Universitaire de Psychiatrie, Centre d’Excellence Thérapeutique-Institut de Psychiatrie, Centre Hospitalier du Rouvray, Sotteville-lès-Rouen, France; ^2^Centre Régional de Psychotraumatologie, Centre Hospitalier Régional Universitaire (CHRU) de Tours, Tours, France; ^3^INSERM U1253 Imagerie et Cerveau (iBrain), Tours, France; ^4^Centre Hospitalier Universitaire (CHU) de Rouen, Rouen, France; ^5^Faculté de Médecine, Normandy University, Rouen, France; ^6^Service Hospitalo-Universitaire, Pôle Hospitalo-Universitaire Psychiatrie Paris 15, Groupe Hospitalier Universitaire Paris, Paris, France; ^7^Université Paris Cité, Paris, France; ^8^Unité de Recherche Clinique (URC), EPS Ville Evrard, Neuilly-sur-Marne, France

**Keywords:** Esketamine, treatment-resistant depression, post-traumatic stress disorder, assisted-therapy, trauma-focused psychotherapy

## Abstract

**Introduction:**

Major depressive disorder (MDD) is more likely to resist to usual treatment when it is associated with post-traumatic stress disorder (PTSD). Capitalizing on the effect of ketamine in both treatment-resistant depression (TRD) and PTSD, we conducted a study in order to assess the efficacy of intranasal (IN) Esketamine in patients having TRD with comorbid PTSD.

**Materials and Methods:**

In this open-label, single arm, retrospective pilot study, 11 patients were treated with IN Esketamine (56 or 84 mg) with a longitudinal follow-up of 6 months. IN Esketamine was administered twice weekly during the first month, once weekly during the second month, and then once every 1 or 2 weeks. Patients were assessed with Montgomery-Åsberg Depression Rating Scale (MADRS), Patient Health Questionnaire 9 items, Global Assessment of Functioning (GAF), and Clinical Global Impression-Suicide Scale (CGI-SS).

**Results:**

We included 9 women and 2 men (mean age 47.3 ± 11.1 years). The mean (SD) MADRS scores decreased significantly from 38.6 (6.4) at baseline to 18.2 (10.03) after 6 months of IN Esketamine; 7 patients were responders and 3 patients were in remission. The percentage of patients who were moderately to severely suicidal declined from 63.6% at baseline to 27.3% after 1 month of IN Esketamine sessions. No serious adverse reactions were observed.

**Conclusion:**

This study reports the outcomes of 11 severely ill patients with comorbid TRD and PTSD after IN Esketamine treatment. Esketamine significantly improved depression symptoms, suggesting that it is likely to be a treatment of choice in this specific population.

## Introduction

Major depressive disorder (MDD) is a common psychiatric disorder and is considered as one of the leading causes of disability worldwide (1). More than one third of depressed patients fail to fully respond to antidepressant treatments at adequate doses and duration, and are regarded as treatment-resistant depression (TRD) patients (2). Treatment resistance is characterized by an absence of symptomatic remission after the use of two successive trials of antidepressants of different pharmacological classes, well conducted in terms of dosage and duration while ensuring quality compliance (3, 4). A wide range of sociodemographic (female sex, age, financial insecurity, low level of education, etc.) and clinical factors, such as psychiatric and somatic comorbidities, are associated with treatment resistance (3). Post-traumatic Stress Disorder (PTSD) is one of those comorbidities (5, 6). It is a chronic and disabling condition arising after exposure to a severe traumatic event, characterized by persistent reexperiencing, avoidance, and hyperarousal symptoms. Risk for PTSD depends on trauma exposure severity, cumulative number of traumas, and trauma type; interpersonal traumas (physical and sexual assault in the context of relationship) carrying the highest risk (7). Patients with comorbid depression and PTSD have greater functional impairment (8) and their likelihood of suicidality is increased by more than three times compared to individuals with only one of these disorders (9).

Treatment strategies for TRD include antidepressants [e.g., selective serotonin reuptake inhibitors (SSRIs), serotonin-norepinephrine reuptake inhibitors (SNRIs), tricyclics and monoamine oxidase inhibitors], psychotherapy and brain stimulation techniques [e.g., electroconvulsive therapy (ECT) and repetitive transcranial magnetic stimulation (rTMS)] (10). Because some patients do not respond to those treatments, additional therapeutic strategies are strongly needed (11). In recent years, a growing body of evidence has implicated the glutamatergic system in the pathogenesis of depression, *N*-methyl-D-aspartate (NMDA) glutamate receptors being identified as a potential pharmacotherapeutic target for MDD, including TRD (12–14). Intravenous ketamine, a non-competitive receptor antagonist of NMDA glutamate receptors, was found to exhibit a robust and rapid onset of efficacy in patients with TRD when administered at subanesthetic doses (0.5 mg/kg) (15–19). Glutamate is also involved in stress responsivity, the formation of traumatic memories, and the pathophysiology of PTSD (20). So ketamine was proposed as a potential treatment for chronic PTSD (21–23) or for comorbid PTSD with TRD (24), although the results regarding ketamine efficacy in this indication are contradictory (25).

Nevertheless, ketamine is known for its abuse potential and profound adverse effects, such as psychotomimetic symptoms, neurotoxicity, cognitive impairment, and hypertension. These effects appear to be less frequent with its S-enantiomer or Esketamine, making it preferable to use (26). Oral Esketamine administration yields a low bioavailability of around 20%, which stimulated the development of its intranasal (IN) form (27). Indeed, the bioavailability and the kinetics of effects of ketamine vary considerably according to the route of administration (e.g., bioavailability: oral: 20%; intramuscular: 90%; rectal: 25%; intranasal: 50%; epidural: 77%; kinetics of effects: oral: delay 15–30 min, duration: 60–90 min; intramuscular: delay: 10–15 min, duration: 30–120 min; intravenous: delay: 1–2 min, duration: 20–60 min) (28). Granted marketing authorization by the European Medicines Agency (EMA) for the treatment of TRD in December 2019 (29), Esketamine nasal spray is used as an antidepressant for TRD. It delivers a 28 mg Esketamine dose *via* two sprays (one per nostril) (29, 30). Phase-3 short-term trials of Esketamine nasal spray (28, 56, or 84 mg) plus an oral antidepressant have demonstrated a statistically significant reduction in depressive symptoms [reduction from baseline Montgomery–Åsberg Depression Rating Scale (MADRS) total score] in patients with TRD compared with an oral antidepressant plus placebo nasal spray (31), and a sustained decreased risk of relapse among stable remitters and responders in long-term trials (29, 32, 33). Long-term safety data showed that most treatment-emergent adverse events (AEs) were mild or moderate in intensity, and resolved on the same day (33). However, the optimum dose, duration, and frequency of use are not fully understood yet (34) and potential indications still need to be clarified. There is no head-to-head data to compare ketamine or Esketamine formulation in terms of tolerance or efficacy. Nevertheless, a recent case series demonstrated that 10 consecutive patients who had responded to IV racemic ketamine for TRD successfully maintained their antidepressant response when switched to IN Esketamine (35).

On the basis of the reported efficacy of intravenous ketamine on PTSD and TRD, we hypothesized that IN Esketamine could be effective in TRD patients with comorbid PTSD. This study is the first one to examine the efficacy and the safety of repeated IN Esketamine administration over a 6-month period on symptoms of depression in this specific population. We further discuss the possible mechanisms of action and the potential synergistic effect with psychotherapeutic intervention.

## Materials and Methods

### Participants

We have led an open-label, single arm, retrospective pilot study on 11 adult patients (aged 18–65 years) who have received from Esketamine nasal spray between February 2020 and November 2021 in one psychiatric department specialized in TRD. Patients underwent a psychiatric evaluation by a board-certified psychiatrist (MR or CB) to confirm diagnosis of major depression and comorbid PTSD according to the criteria in the Diagnostic and Statistical Manual of Mental Disorders, Fifth Edition (DSM-5). All participants met the criteria for TRD (3) and PTSD (DSM-5). Written informed consent was obtained from all participants before participation. Under French ethical law (public Health code), retrospective studies based on the exploitation of routine care data do not have to be submitted to an ethics committee. This research was performed in accordance with the Declaration of Helsinki.

### Procedures

All participants received Esketamine nasal spray co-administered with a newly initiated oral antidepressant in either a full or partial hospitalization setting, as recommended by the Summary of Product Characteristics (SmPC) approved by ANSM [European Medicines Agency (36)]. All patients had baseline blood tests and an electrocardiogram to determine medical stability before the initiation of Esketamine. Post-administration monitoring was undertaken by a healthcare professional for ≥120 min. Throughout their treatment with Esketamine, the patients benefited from individualized follow-up by the same referring nurse. Esketamine was initiated at 56 mg and adjusted on an individual basis during the treatment period in accordance with the following treatment guidelines (29, 36): Weeks 1–4: 56 or 84 mg twice weekly; Weeks 5–8: 56 or 84 mg once weekly; and Week 9 and onward: 56 or 84 mg once every 1 or 2 weeks (Supplementary Figure 1). However, psychiatrists were free to adjust treatment dosage and frequency on an individual basis, depending on the patient’s response and tolerance to treatment. During Esketamine treatment, voluntary patients were offered trauma-focused psychotherapy [cognitive behavioral therapy (CBT) or eye movement desensitization and reprocessing (EMDR)] once every 1 or 2 weeks. Psychotherapy sessions took place within one week of Esketamine administration.

### Measures

Scores were collected every month. The primary outcome was changes in depression score, assessed with the MADRS, between treatment initiation at baseline (Day 1) and 6 months of treatment (M6). A MADRS score of 30 is considered a definition of severe depression (37). At 6 months, patients who achieved a 50% or greater reduction in their MADRS scores were considered as responders, while patients who obtained a MADRS score inferior to 12 were remitters. Secondary outcomes included changes in scores on Patient Health Questionnaire-9 (PHQ-9), Global Assessment of Functioning (GAF) and Clinical Global Impression-Suicide Scale (CGI-SS) between baseline, and M6. Side effects and tolerability were assessed after each Esketamine administration. We also assessed PTSD symptoms as an exploratory variable in patients who were undergoing psychotherapy using the PTSD Checklist for DSM-5 (PCL-5) between psychotherapy initiation and the 6th month of psychotherapy (38). A PCL-5 score of 32 was deemed to have the greatest likelihood of correctly categorizing a participant as having PTSD as per the DSM-5 guidelines (38).

### Statistical Analysis

Changes in quantitative outcome measures from the baseline to 6 months after Esketamine initiation were examined using non-parametric Friedman tests, due to the small sample size. Statistical analyses were conducted using SPSS, version 28 (IBM, Armonk, NY, United States).

## Results

### Sample Characteristics

As shown in Supplementary Table 1, we included 9 female and 2 male patients with comorbid TRD and PTSD (mean age 47.27 ± 11.07 years, range 24–59). The majority of patients were in a relationship (*n* = 8), on sick leave (*n* = 5) or unemployed (*n* = 4). Nine patients (82%) have already attempted suicide in their lifetime. Patients suffered from chronic PTSD related to rape (*n* = 2), sexual abuse in childhood (*n* = 4) or other traumatic experiences (one suicide of family member, one brutal love breakup, and three workplace bullying). Nine patients also had other comorbidities, namely, anxiety disorders (*n* = 4), chronic pain (*n* = 3), addiction (*n* = 1), and eating disorder (*n* = 1).

### Psychotropic Treatment

Treatment with IN Esketamine was initiated at 56 mg, followed by a titration up to a target dose of 84 mg. At 6 months, all patients were still receiving a dose of 84 mg per session except for one patient who was receiving 56 mg due to poor tolerance (nausea). However, the rhythm of the sessions was conducted in accordance with the recommended Esketamine administration protocol for all patients. The mean number of Esketamine sessions administered in 6 months were 25.0 (5.3) ranging from 13 to 28. Concomitant medication prescriptions included SSRIs (*n* = 2), serotonin/norepinephrine reuptake inhibitors (*n* = 7), α2 antagonists (*n* = 1), tricyclics (*n* = 1), atypical antipsychotics (*n* = 9), mood stabilizers (*n* = 10), and benzodiazepines (*n* = 6). Treatment (other than Esketamine) was not changed during the follow-up period.

To test the effect of concomitant antidepressant treatments on our primary outcome (change in MADRS between baseline and 6 months of Esketamine treatment), we converted antidepressant treatments into fluoxetine equivalent (39). No significant correlation (ρ = –0.598, *p* = 0.068) was found between relative improvement (between baseline and 6 months of esketamine) and antidepressants dose (fluoxetine-equivalents).

### Evolution of Symptoms

**FIGURE 1:**
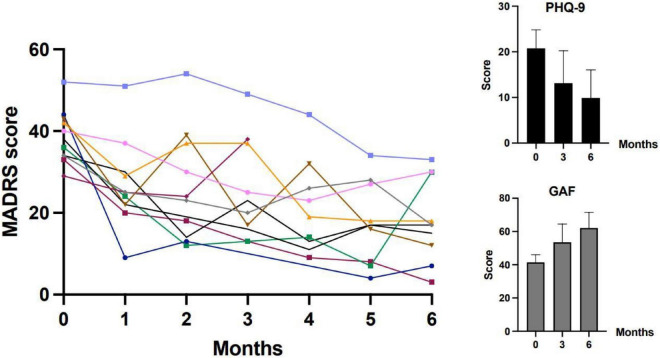
The evolution of depressive symptoms (through MADRS and PHQ-9) and global functioning (through GAF) during treatment. MADRS, Montgomery-Åsberg Depression Rating Scale; PHQ-9, Patient Health Questionnaire-9; GAF, Global Assessment of Functioning. The main figure (on the left) shows MADRS scores for individual participants. The two figures on the right report means and standard deviations for PHQ-9 and GAF.

At the baseline, the mean Maudsley staging score was 10.4 (1.6) (range: 7–12), with moderate (*n* = 5) to severe (*n* = 6) level of resistance (40). The evolution of depressive symptoms (through MADRS and PHQ-9) and global functioning (through GAF) are illustrated in Figure 1. The mean MADRS score significantly decreased during the treatment period (–13.5 points at 3 months and –20.4 at 6 months, *p* < 0.001, see Table 1). As reported in Table 1, the PHQ-9 scores significantly decreased over the treatment period (*p* = 0.012), whereas the GAF scores significantly increased (*p* = 0.001). The number of patients who achieved response (defined by a reduction of at least 50.0% of the MADRS total score) increased with time from one (9.1%) after one month of treatment, to five (45.5%) after 3 months and seven (63.6%) after 6 months. Regarding remission (defined as a MADRS total score ≤ 12), three patients (27.3%) reached remission at 3 and 6 months [including one (9.1%) who achieved remission one month after treatment initiation]. One patient did not respond to treatment and stopped after 25 sessions.

**TABLE 1:** Evolution of mood and functioning outcomes during treatment.

Mean (SD)	MADRS		PHQ-9		GAF		CGI-SS	
Baseline	38.6 (6.4)	*n* = 11	20.8 (4.1)	*n* = 08	41.5 (4.6)	*n* = 11	2.8 (1.0)	*n* = 11
1 month	26.7 (10.6)	*n* = 11	14.9 (5.3)	*n* = 09	54.3 (10.3)	*n* = 08	2.3 (1.0)	*n* = 11
2 months	25.7 (13.1)	*n* = 11	14.1 (5.5)	*n* = 09	52.0 (10.0)	*n* = 08	1.9 (0.9)	*n* = 10
3 months	25.1 (12.3)	*n* = 10	13.1 (7.1)	*n* = 08	53.5 (11.0)	*n* = 08	1.9 (1.0)	*n* = 10
4 months	21.2 (11.4)	*n* = 09	9.3 (4.3)	*n* = 06	57.6 (8.7)	*n* = 07	1.8 (1.0)	*n* = 08
5 months	17.6 (9.8)	*n* = 10	12.1 (8.5)	*n* = 08	63.9 (11.7)	*n* = 09	1.4 (0.7)	*n* = 10
6 months	18.2 (10.0)	*n* = 10	9.9 (6.2)	*n* = 08	62.1 (9.5)	*n* = 10	1.7 (1.1)	*n* = 10
*p*-value	*p* < 0.001		*p* = 0.012		*p* = 0.001		*p* = 0.060	

*Mean (standard deviation) and sample size (n) are reported. P-values are results of Friedman tests. MADRS, Montgomery-Åsberg Depression Rating Scale; PHQ-9, Patient Health Questionnaire-9; GAF, Global Assessment of Functioning.*

As for suicidality, the percentage of patients who were at least moderately to severely suicidal (CGI-SS score ≥ 3) went from 63.6% before treatment to 27.3% after 1 month of Esketamine treatment, and then to 20% after 3 months. However, the decrease was not statistically significant (Table 1).

### Evolution of Post-traumatic Symptoms Under Psychotherapy

Seven patients received psychotherapy in parallel with the administration of IN Esketamine: either cognitive and behavioral therapy focused on trauma (*n* = 3), or EMDR (*n* = 1) or both (*n* = 3). Psychotherapy occurred on average (SD) 3.6 (2.8) months after starting Esketamine treatment. Their post-traumatic symptoms were assessed through the PCL-5 before and after psychotherapy with a mean interval of 5.6 (5.5) months. The mean score went from 58.6 (3.9) before treatment to 32.7 (16.0) after treatment. The mean (SD) relative improvement was 45.3% (25.5).

### Discontinuation Rates

Among the eleven patients included in the analyses, three patients stopped IN Esketamine before 6 months: one patient stopped 2 months after Esketamine initiation (i.e., 13 sessions) because of remission, one patient stopped after 4 months (16 sessions) due to travel difficulties and a third patient stopped due to lack of efficacy of IN Esketamine (25 sessions) and started treatment with a non-selective monoamine oxidase inhibitor.

### Adverse Effects and Tolerability

The most frequent AEs were dissociation (*n* = 7), somnolence (*n* = 4), nausea (*n* = 4), sedation (*n* = 3), dizziness (*n* = 3), anxiety (*n* = 2), and increased blood pressure or hypertension (*n* = 1). Most side effects were moderate and did not require discontinuation of treatment. One patient had nausea and required anti-emetic treatment before sessions. For this patient, the increase of Esketamine dosage to 84 mg had to be delayed. The dissociative effects consisted of disinhibition with verbalization of traumatic events (*n* = 4) or derealization (*n* = 3). We did not observe any serious adverse effects throughout the study. In particular, no safety issue due to repeated Esketamine administration occurred in our study.

## Discussion

This pilot study provides the first data evaluating IN Esketamine treatment in patients with dual diagnosis of TRD and PTSD, suggesting that this treatment, in association with antidepressants and with psychotherapy, could rapidly reduce depressive symptoms in this population with a long-lasting effect up to 6 months. In our sample, two thirds of TRD patients achieved response and one third reached remission after 6 months of IN Esketamine treatment. The risk of suicide was importantly reduced, although not statistically significant: the percentage of moderately to severely suicidal patients was divided by 3 in 6 months of treatment. PTSD symptoms have also importantly improved for patients that had both Esketamine and trauma-focused therapy, with a mean (SD) reduction of 45.3% (25.5) of PCL-5 after 6 months of therapy. This latter result is exploratory and only concerns a sub-group of patients but seems clinically meaningful considering the severity, the resistance and the functional outcome of the disease presented by our population.

In previous studies, Esketamine has been shown to be effective in TRD. In our study, we found that patients with comorbid TRD and PTSD have similar to higher rates of response than patients with TRD alone in previous studies [63.6% in our study versus 45.6, 52.2, and 61.4% in TRANSFORM-1 (41) and TRANSFORM-2 (31) studies]. However, they have lower rates of remission (27.3% in our study versus 33.3, 34.8, and 46.5% in TRANSFORM-1 and TRANSFORM-2 studies). This difference may be explained by the persistence of residual symptoms that could be specific to the PTSD comorbidity. Several studies found that racemic ketamine could decrease PTSD symptoms (21–23, 42) but this effect was recently challenged (25). Still, the only study that previously explored the efficacy of racemic ketamine on comorbid TRD and PTSD found a quick and significant reduction of symptoms, but with a limited effect over time (median time to relapse of 41 days) (24). Our study therefore confirms these results with IN Esketamine.

This action of Esketamine in patients with both depressive and PTSD symptoms might be explained by common pathophysiological features. Indeed, in both depression and PTSD, patients are more sensitive to negative emotional stimuli which trigger an increased activation of amygdala and anterior cingulate cortex and a decreased activation of the prefrontal cortex (43, 44). Interestingly, low doses of ketamine have been shown to reverse that pattern in patients with MDD (45). On the other hand, PTSD is a risk factor for TRD (5, 6) and patients with TRD perceive their onset-related events as serious psychological distress symptoms (46). It was also shown that patients with TRD who have experienced traumatic events are relieved by trauma-focused psychotherapy (47), so one could postulate that part of the efficacy of ketamine on TRD may be related to a beneficial action on underlying traumas. Finally, ketamine facilitates fear memory extinction (48, 49) which could help patients with PTSD to have less avoidance behaviors and to enter in a psychotherapeutic process. The main mechanism of action of Esketamine is to block NMDA receptors on GABA interneurons and to activate the alpha-amino-3-hydroxy-5-methyl-4-isoxazolepropionic acid (AMPA) receptors. This action might increase neurotrophic signaling, thereby restoring synaptic function and improving neural plasticity and synaptogenesis (50) in particular in the prefrontal cortex (51, 52). Ketamine reverses structural and functional deficits induced by chronic stress exposure (53), which could help both patients with PTSD and with depression. Indeed, in individuals with chronic PTSD, persistent, recurrent intrusions could represent a form of chronic stress that prolongs and worsens maladaptive biological changes, including synaptic atrophy, dysregulation of glutamatergic transmission, and disrupted connectivity in corticolimbic circuitry (54, 55). Finally, ketamine normalizes the disrupted connectivity between the prefrontal cortex, the default mode network and other key brain regions, that is observed in depression (56, 57) and could similarly restore cerebral connectivity in these regions in PTSD (58).

Importantly, the present study also demonstrated that repeated IN Esketamine sessions were safe and well-tolerated, with transient dissociative symptoms that disappeared within 2 h. Indeed, over 6 months, we did not observe any serious adverse effects. These data are consistent with previous ecological observations which did not identify new safety signals (59) compared to the initial data that allowed Esketamine approval. This is a crucial result since previous studies suggested that Esketamine could trigger higher dissociative symptoms than racemic ketamine when administered in acute trauma phases (60). On the contrary, the dissociative effects could induce a surprisingly positive impact. Indeed a trance state, as described during ketamine-assisted psychotherapy (61) was observed in our study for 6 patients (54.5%). It promoted communication and allowed some of them to verbalize their traumatic experience for the first time. Therapists then observed that these patients were getting rid of fear more easily, enabling them to create new positive associations. Such an effect could have directly emerged from the disinhibition and the fear extinction induced by ketamine (48, 49) or from an increased neuroplasticity (51, 52) that could have reduced the rigidity of traumatic memories, making them finally accessible to psychotherapy. Ketamine-assisted psychotherapy has shown to be a relevant approach in other chronic, severe or resistant diseases, e.g., in alcohol use disorder (62), heroin addiction (63), and TRD without comorbid PTSD (64). Previous articles developed clinical guides of ketamine-assisted psychotherapy (65, 66). However, its modalities, notably in the treatment of PTSD, remain to be better defined. More specifically, the optimal moment to start psychotherapy and how it should be articulated with Esketamine sessions in order to have the most benefit should be specified. Here we tentatively suggest that the first sessions may allow the patients to get used to subjective changes induced by Esketamine and to benefit from early anxiolytic and antidepressant effects. In this sense, it could be more appropriate to start trauma-focused psychotherapy a bit later. In contrast, patients could be accompanied before and during the first Esketamine sessions with techniques that have been developed to improve tolerance and avoid “bad-trip” during psychotropic-assisted therapy (67).

Despite encouraging results, our study had several limitations, notably the small number of patients included and the absence of sample size calculation. The open-label design and the absence of placebo control does not allow us to firmly conclude in terms of efficacy. In addition, there is an overlap between depressive symptoms and some PTSD symptoms and our study cannot accurately describe the respective effect of Esketamine on each of them or tease apart the respective contributions of the pharmacological and the psychotherapeutic treatment in the observed improvement. Moreover, we cannot eliminate a confounding effect of different drugs or other comorbid disorders since there was no control group. Quantitative measures of the intensity of dissociation during Esketamine administration would have allowed us to explore whether it was predictive of response or remission of PTSD symptoms. In the future, the efficacy of Esketamine-assisted therapy may be further assessed with a more structured protocol including a control group.

## Conclusion

This pilot study is the first one to assess Esketamine efficacy on comorbid TRD and PTSD. Our results suggest rapid and sustained effects of Esketamine both on depressive and PTSD symptoms at 6 months even if patients were severely ill and importantly disabled by their disorders. The properties of Esketamine seem to combine particularly well with trauma-focused therapies and those two approaches probably have a synergistic effect. Esketamine could therefore be a treatment of choice in TRD patients with comorbid PTSD.

## Data Availability Statement

The raw data supporting the conclusions of this article will be made available by the authors, without undue reservation.

## Ethics Statement

Ethical review and approval was not required for the study on human participants in accordance with the local legislation and institutional requirements. The patients/participants provided their written informed consent to participate in this study.

## Author Contributions

MR, WE-H, OG, RG, VM, and LB conceived and conceptualized the study. MR, ChB, CaB, and DR collected data. VM and DR performed formal analysis of data collected. MR and VM wrote the preliminary draft. WE-H and LB revised and edited the final draft. All authors reviewed and approved the final draft of the manuscript and made substantial contributions to this study.

## Conflict of Interest

MR has received honoraria or consulting fees from Janssen-Cilag and Lundbeck-Otsuka. WE-H reports personal fees from Air Liquide, Chugai, Lundbeck, Janssen-Cilag, Otsuka, and UCB. LB has participated to a Medical Education Steering Committee for Janssen-Cilag. OG has received honoraria or consulting fees from EISAI, Lundbeck, Otsuka/Lundbeck, Janssen-Cilag, MAGPREP, and Bioprojet. RG has received compensation as a member of the scientific advisory board of Janssen, Lundbeck, Roche, SOBI, and Takeda. He has consulted and/or served as speaker for Astra Zeneca, Boehringer-Ingelheim, Pierre Fabre, Lilly, Lundbeck, MAPREG, Otsuka, Pileje, SANOFI, Servier, LVMH, and has received research support from Servier. Co-founder and stock shareholder: Regstem. The remaining authors declare that the research was conducted in the absence of any commercial or financial relationships that could be construed as a potential conflict of interest.

## Publisher’s Note

All claims expressed in this article are solely those of the authors and do not necessarily represent those of their affiliated organizations, or those of the publisher, the editors and the reviewers. Any product that may be evaluated in this article, or claim that may be made by its manufacturer, is not guaranteed or endorsed by the publisher.
