# Supplementary material for: Efficacy and Safety of Intranasal Esketamine in Patients With Treatment-Resistant Depression and Comorbid Chronic Post-traumatic Stress Disorder: Open-Label Single-Arm Pilot Study
Source: Front Psychiatry. 2022 Jul 8;13:865466. doi: 10.3389/fpsyt.2022.865466 (PMC9305073; doi:10.3389/fpsyt.2022.865466)
Supplement: Supplementary file 1 [file Data_Sheet_1.doc]

**Supplementary Figure 1: Experimental design.**

| **TIMEPOINT**** | ***Baseline*** | ***M1*** | ***M2*** | ***M3*** | ***M4*** | ***M5*** | ***M6*** | ***M7-M9*** | ***After psychotherapy*** |
| --- | --- | --- | --- | --- | --- | --- | --- | --- | --- |
| **ESKETAMINE:** | Twice a week | | Once a week | Once every 1 or 2 weeks | | | |  |  |
| *Sessions* | ♦♦♦♦♦♦♦♦ | | ♦♦♦♦ | ♦♦♦♦  or ♦♦ | ♦♦♦♦  or ♦♦ | ♦♦♦♦  or ♦♦ | ♦♦♦♦  or ♦♦ |  |  |
| *Dose* | 56 or 84 mg | | | | | | |  |  |
| ***PSYCHOTHERAPY***  ***(optional)*** | - | | - | Once every 1 or 2 weeks | | | | | |
| *Sessions* | - | | - | ♦♦♦♦  or ♦♦ | ♦♦♦♦  or ♦♦ | ♦♦♦♦  or ♦♦ | ♦♦♦♦  or ♦♦ | ♦♦♦♦  or ♦♦ by month |  |
| ***Clinical scales***  *MADRS*  *PHQ-9*  *GAF*  *CGI-SS*  *PCL-5* | x  x  x  x | x  x  x  x | x  x  x  x | x  x  x  x  **x** | x  x  x  x | x  x  x  x | x  x  x  x |  | **x** |

Notes. MADRS = Montgomery-Åsberg Depression Rating Scale, PHQ-9 = Patient Health Questionnaire-9, GAF = Global Assessment of Functioning.

**Supplementary Table 1: Socio-demographic and clinical characteristics of each patient, treatments and side-effects.**

| **Patient** | **Sex** | **Age (years)** | **Marital Status** | **Level of Education (years)** | **Employment Status** | **Comorbidity** | **Traumatic event** | **Drugs/day** | **Therapy** | **Side effects** | **MADRS Relative improvement*** |
| --- | --- | --- | --- | --- | --- | --- | --- | --- | --- | --- | --- |
| **1** | F | 37 | in couple | 11 | on sick leave | chronic pain | rape | clomipramine 250mg, mianserine 60mg, risperidone 0,5 mg, lamotrigine 200mg, diazepam 8mg | CBT | dissociation | 50 |
| **2** | F | 59 | single | 11 | employed | none | sexual abuse in childhood | venlafaxine 225mg, lithium LP 800mg, quetiapine 300mg | CBT then EMDR | dissociation, somnolence | 57.1 |
| **3** | M | 46 | married | 12 | unemployed | THC abuse | sexual abuse in childhood | venlafaxine 375mg, mirtazapine 45mg, lithium LP 800mg, quetiapine 150mg, lamotrigine 200mg; diazepam 20mg | CBT | sedation, dissociation, increased blood pressure | 25 |
| **4** | F | 54 | single | 11 | on sick leave | chronic pain | suicide of family member | venlafaxine 225mg, mirtazapine 45mg, lithium LP 800mg, quetiapine 300mg | no | somnolence, dissociation, nausea, dizziness | Stop before 6 months |
| **5** | M | 35 | in couple | 11 | unemployed | social phobia | sexual abuse in childhood | sertraline 200mg, lamotrigine 200mg, risperidone 1mg | CBT then EMDR | dissociation, nausea | 72.1 |
| **6** | F | 59 | married | 11 | retired | chronic pain | sexual abuse in childhood | venlafaxine 75mg, lithium 600mg, alprazolam 1.5mg | no | somnolence, anxiety | 84.1 |
| **7** | F | 52 | married | 13 | on sick leave | obesity | workplace bullying | venlafaxine LP225mg, lithium LP 800mg, lamotrigine 200mg, mirtazapine 30mg, lozapepam 3 mg | CBT then EMDR | dissociation, dizziness | 36.5 |
| **8** | F | 50 | in couple | 15 | unemployed | anxiety disorder | brutal love breakup | clomipramine 200mg, lithium LP 800mg, lorazepam 7.5mg | no | somnolence, nausea, dizziness | 60.5 |
| **9** | F | 56 | in couple | 11 | unemployed | anxiety disorder | workplace bullying | sertraline 150mg, quetiapine 150mg, lorazepam 5mg, hydroxyzine 75mg | no | sedation, nausea, dissociation | 16.7 |
| **10** | F | 48 | married | 14 | on sick leave | social phobia | workplace bullying | venlafaxine LP300mg, mirtazapine 30mg, lithium LP 1000 mg, pramipexole 2,1mg | EMDR | nausea and vomiting, anxiety, sedation | 50 |
| **11** | F | 24 | single | 16 | on sick leave | none | rape | Venlafaxine LP225mg, lithium LP 800mg, lamotrigine 200mg | CBT | none | 90.9 |

Legend: F: female; M: male; CBT: cognitive and behavioral therapy focused on trauma; EMDR: Eye Movement Desensitization and Reprocessing; *MADRS baseline-6 months/MADRS baseline*100.
